# Endothelial Markers in Type 2 Diabetic Patients with Acute Decompensated Heart Failure: A Pilot Study

**DOI:** 10.3390/metabo15020091

**Published:** 2025-02-03

**Authors:** Martin Jozef Péč, Jakub Jurica, Tomáš Bolek, Ingrid Škorňová, Monika Péčová, Marek Cingel, Simona Horná, Lucia Stančiaková, Ján Staško, Štefan Tóth, Juraj Sokol, Peter Galajda, Marián Mokáň, Matej Samoš

**Affiliations:** 1 Department of Internal Medicine I, Jessenius Faculty of Medicine in Martin, Comenius University in Bratislava, Kollarova 2, 03659 Martin, Slovakia; pec5@uniba.sk (M.J.P.); kubo.jurica@gmail.com (J.J.); ato.bolek@gmail.com (T.B.); marek.cingel1@gmail.com (M.C.); horna.simona@gmail.com (S.H.); peter.galajda@uniba.sk (P.G.); mokanmarian@gmail.com (M.M.); 2 National Centre of Hemostasis and Thrombosis, Department of Hematology and Blood Transfusion, Jessenius Faculty of Medicine in Martin, Comenius University in Bratislava, 03659 Martin, Slovakia; inkaskornova@gmail.com (I.Š.); kucerikovam@gmail.com (M.P.); lstanciakova@gmail.com (L.S.); jan.stasko@uniba.sk (J.S.); juraj.sokol@uniba.sk (J.S.); 3 Department of Oncology, University Hospital in Martin, 03659 Martin, Slovakia; 4 Department of Gerontology and Geriatrics, Faculty of Medicine, P.J. Šafarik University in Košice, 04011 Košice, Slovakia; stefan.toth@upjs.sk; 5 Division of Acute and Interventional Cardiology, Department of Cardiology and Angiology II, Mid-Slovakian Institute of Heart and Vessel Diseases (SÚSCCH, a.s.) in Banská Bystrica, 97401 Banská Bystrica, Slovakia

**Keywords:** type 2 diabetes, endothelial markers, acute decompensated heart failure, diabetic heart disease

## Abstract

Background: Impaired endothelial function has been associated with vascular complications in type 2 diabetes (T2D), but its role in T2D-related heart failure (HF) remains indeterminate. The aim of this study was to assess selected markers of endothelial function in T2D patients with acute decompensated HF. Methods: A pilot prospective study on patients with acute decompensated HF requiring in-hospital admission was carried out. The vascular endothelial growth factor (VEGF), intercellular adhesion molecule 1 (ICAM-1), and vascular cell adhesion molecule 1 (VCAM-1) were assessed at admission and after decongestion. Subsequently, differences in these markers between T2D and non-diabetic (ND) patients were studied. Results: In total, 39 patients (21 with T2D and 18 ND patients) were enrolled. Twenty-eight patients presented with preserved ejection fraction (EF), and 11 presented with reduced EF. Looking at the VEGF levels in T2D patients, on admission, a median of 233.0 pg/mL (1.7–598 pg/mL) was found compared to 106.0 pg/mL (1.7–888 pg/mL) in ND individuals; the differences reached statistical significance (*p* = 0.04). There were no significant differences in VEGF levels after decongestion, and in VCAM-1 (2237 ± 1195 vs. 2699 ± 1093 ng/mL, *p* = 0.37) and ICAM-1 (596 ± 268 vs. 638 ± 437 ng/mL, *p* = 0.79) levels between T2D and ND patients upon admission and after decongestion. The value of EF (preserved or reduced) affected the VEGF levels upon admission. Conclusions: This study identified significantly higher VEGF levels upon admission due to acute decompensated HF in T2D patients.

## 1. Introduction

Impaired endothelial function (endothelial dysfunction) has been associated with chronic vascular (micro- and macrovascular) complications in type 2 diabetes and endothelial markers (EMs) can be used to identify the presence of this condition [1]. For example, vascular endothelial growth factor (VEGF) levels have been associated with diabetic retinopathy [2], diabetic kidney disease (diabetic nephropathy) [3,4], and T2D-related coronary heart disease [5]. In addition, the same association was previously reported for vascular cell adhesion molecule 1 (VCAM-1) and intercellular adhesion molecule 1 (ICAM-1) [6,7,8,9].

More recently, a significant discussion on T2D-related heart failure (HF) has started, and it seems that this disease is one of the leading causes of mortality in patients with T2D [10]. This discussion is still ongoing. Additionally, acute worsening (acute decompensation) of (chronic) HF with the need for patient hospitalization is undoubtedly associated with a worse prognosis [11]. The number of HF-related in-hospital stays strongly predicts the all-cause mortality of HF patients. The median survival was 2.4 after the first admission and just 0.6 years after the fourth in-hospital stay in community HF patients [11]. This surely advocates the need for clinical research on acute decompensated HF.

Furthermore, endothelial dysfunction plays an important role in the development and progression of diabetic cardiomyopathy and T2D-related HF [12]. Increased blood glucose and insulin levels and decreased tissue response to insulin (insulin resistance) lead to impaired endothelial function, including endothelial barrier dysfunction, reduced nitric oxide activity, excessive oxidative stress, and the disrupted regulation of inflammation, leading to an increased subclinical inflammatory reaction. Subsequently, endothelial dysfunction promotes the impaired metabolism of cardiomyocytes, intracellular calcium metabolism dysregulation, endoplasmic reticular stress, mitochondrial dysfunction, the accumulation of advanced glycation end-products, the creation of extracellular matrix deposits leading to increased cardiac stiffness, and increased cardiac fibrosis, which are all strongly related to impaired cardiac systolic and diastolic function and HF development. The role of assessing EM plasma levels as a means of predicting the prognosis of acute decompensated HF remains unclear; however, a previous study showed that decreased VEGF levels are associated with a more severe degree of congestion and an unfavorable clinical outcome in HF patients [13]. In addition, low levels of VEGF independently predict two-year cardiovascular and all-cause mortality in HF patients with high NT-proBNP levels [14]. One suggested mechanism is the impact of (decreased) VEGF levels on the dysfunction of myocardial microcirculation (probably due to the limited formation of collateral circulation) [15]. A previous randomized clinical study showed a positive effect of additional physiological ischemic training on patients with ischemic cardiomyopathy. The authors of this study suggest that the mechanism of this observation could be an improvement in the cardiac blood flow reserve due to the increased VEGF and NO blood levels [16]. Although the mechanism of the role of VEGF in HF is not fully elucidated, these previous observations suggest the possible role of VEGF in the development and progression of HF. For VCAM-1 and ICAM-1, previous studies showed higher levels of these markers in patients with advanced HF [17] and increased endothelial monocyte adhesion, which correlated with an increased expression of VCAM-1 and ICAM-1 in patients with chronic HF [18]. There was also a correlation between these markers and lower left atrial and left ventricular systolic function [19]; moreover, the plasma levels of these markers were significantly affected (decreased) by 10-week-long supervised aerobic training in HF patients [20]. As previously mentioned, all these markers are closely associated with T2D and T2D-related complications [2,3,4,5,6,7,8,9,12]. Furthermore, treatment with empagliflozin led to a significant reduction in VCAM-1 levels and an improvement in cardiac remodeling in an animal model of metabolic syndrome [21]. Nevertheless, there is no study specifically examining the role of EM assessment in individuals with T2D-related HF. Therefore, the aim of this study was to assess selected markers of endothelial function in T2D patients with acute decompensated HF. Due to the aforementioned data and the relationship between endothelial dysfunction and T2D-related HF, we hypothesized that VEGF, VCAM-1, and ICAM-1 could be the best markers for monitoring the severity of acute heart failure (congestion) and the impact of administered therapy on this congestion, and in the changes in the plasma levels of these markers would be more highly expressed (VEGF is expected to be lower, whereas VCAM-1 and ICAM-1 are expected to be higher) in patients with T2D.

## 2. Materials and Methods

### 2.1. Design and Patients

This was a pilot prospective observational study enrolling patients with acute decompensated HF admitted to the Department of Internal Medicine of a tertiary care hospital between May 2023 and August 2024. Patients were enrolled if they met the ESC diagnostic criteria [22] for acute HF and had a history of chronic HF, which is also in line with the ESC HF diagnostic criteria prior to the acute event. Patients with “de novo” HF, a short life expectancy (less than 6 months) due to non-cardiovascular causes (such as active cancer or advanced chronic kidney or liver disease), patients with stage 5 chronic kidney disease requiring hemodialysis, and patients with comorbidities affecting correct T2D diagnosis (such as chronic inflammatory diseases treated with glucocorticoid therapy, acute worsening of chronic obstructive lung diseases requiring glucocorticoid administration, acute infections, etc.) were excluded from this study. The selected exclusion criteria were based on the following rationale. Firstly, this study aimed to assess T2D-related changes in acute decompensated chronic heart failure; therefore, we excluded patients with “de novo” HF as this might also be due to reversible causes (myocardial stunning and acute myocardial inflammation), and these patients might not develop chronic HF. Secondly, we excluded patients with a low life expectancy due to terminal non-cardiovascular diseases and with stage 5 chronic kidney disease requiring hemodialysis as endothelial function might be significantly affected in these patients by their non-cardiovascular disease [23,24,25]. Moreover, the management of de-congestion in patients with hemodialysis is challenging, and diuretic therapy alone usually fails to achieve decongestion in these patients. As hemodialysis itself might affect the levels of endothelial markers, these patients needed to be excluded from our analysis [26]. Finally, patients with comorbidities affecting a correct T2D diagnosis needed to be excluded from the study as it was not possible to confirm/exclude the diagnosis of T2D in these patients and consequently to divide these individuals into a T2D or a non-diabetic sub-group. As this was a pilot study, no sample size calculation was performed, and the size of the sample was determined by the duration of the study. Patients were enrolled subsequently if they fulfilled the study inclusion criteria while not meeting the study exclusion criteria.

Patients with a known and correctly diagnosed T2D [27] were enrolled in the T2D group. Patients with no history of T2D were enrolled in a non-diabetic group if they had normal standard glucose levels and a normal standard glucose tolerance test prior to discharge (fasting plasma glucose < 5.6 mmol/L and plasma glucose two hours after ingestion of 75 g of glucose < 7.8 mmol/L). During their in-hospital stay, all patients received intravenous loop diuretic therapy. There was no standardized protocol for diuretic therapy, as it was at the discretion of the attending physician. Patients with a systolic blood pressure ≥ 110 mmHg received intravenous vasodilators; patients who presented with hypotension and/or tissue hypoperfusion upon admission received intravenous inotropic therapy (dobutamine). The discretion of administering intravenous digoxin and other heart failure therapy (including chronic therapy) was left to the attending physician. After decongestion, patients were switched to oral diuretics, and chronic HF therapy was up-titrated according to the ESC guidelines [22] and local pharmacotherapy policy. A standard echocardiographic study was performed on all the enrolled patients after decongestion on a GE Vivid^®^ 95 (GE HealthCare, Chicago, IL, USA) ultrasound machine. Left ventricular ejection fraction (LVEF) was calculated using the Simpson Bi-plane method with ECG-triggered semiautomatic analysis (AutoEF^®^, GE HealthCare). For this analysis, three cardiac cycles were recorded in apical four- and apical two-chamber projections by an experienced and board-certified examiner. In patients with bad image quality on native echocardiography, 1–2 mL of an ultrasound-enhancing agent (SonoVue^®^, Bracco International B.V., Milan, Italy) was administrated intravenously to increase the opacification of the left ventricle. Left ventricular endocardial contours were traced automatically in the end-diastole and end-systole by ECG-triggering and were adapted manually by the examiner if the automatic analysis misidentified the contour. Subsequently, the automatic volume-based calculation of LVEF was performed by an ultrasound machine. The patients were divided into those with reduced (r) and preserved (*p*) LVEF using a cut-off value of LVEF ≤ 40%.

All enrolled patients signed a written informed consent form prior to blood sampling. After obtaining this written informed consent for study participation, blood samples were taken at admission (sample 1) and after decongestion prior to patient discharge (sample 2) for the assessment of the selected markers of endothelial function. Decongestion was defined as a summary of the following: (1) no detectable clinical symptoms and signs of congestion, (2) no more than 1 B-line per intercostal space on point-of-care lung ultrasound, (3) the normal diameter of inferior vena cava with inspiratory collapse on point-of-care ultrasound, and (4) a significant decrease in NT-proBNP levels. Blood sampling was performed using standard vacutainer blood collection tubes with EDTA as the anticoagulant agent. The plasma levels of VEGF, VCAM-1, and ICAM-1 were assessed in this study. The primary endpoint was to assess the effect of T2D on changing EM plasma levels upon admission and after decongestion. The secondary endpoint was to assess the role of changing EM plasma levels as a marker of decongestion in acute decompensated HF.

### 2.2. Laboratory Assessment of Endothelial Markers

Collected blood samples were centrifuged for 15 min at 1000× *g* within 30 min after collection and stored at −30 °C until the time of laboratory analysis. Samples were examined for the plasma levels of VEGF, VCAM-1, and ICAM-1. The analysis was performed with the Dynex MRW AM60 washer (DYNEX TECHNOLOGIES, Prague, Czech Republic) and Wellcozyme 7000A reader (Dynatech Laboratories, Galena, MO, USA) at a certified tertiary care hospital laboratory of hemostasis and thrombosis.

VEGF plasma levels were assessed using a Quantikine ^®^ Human VEGF immunoassay (R&D Systems^®^, Minneapolis, MN, USA; Catalog Number PDVE00) calibrated against a highly purified Sf 21-expressed recombinant human VEGF165 produced at R&D^®^. The physiological range of VEGF levels was within 62–107 pg/mL, as stated by the manufacturer.

For the assessment of the VCAM-1 level, a “sandwich” enzyme-linked immunoassay, the Quantikine ^®^ Human VCAM-1/CD106 immunoassay (R&D Systems^®^, Minneapolis, MN, USA; Catalog Number PDVC00), was used. The physiological range of VCAM-1 levels was within 341–897 ng/mL, as stated by the manufacturer.

ICAM-1 levels were assessed with a Quantikine ^®^ Human sICAM-1 immunoassay (Biotechne^®^/R&D Systems^®^, Minneapolis, USA; Catalog Number PDCD540). The physiological range of ICAM-1 levels was within 100–307 ng/mL, as stated by the manufacturer.

### 2.3. Statistical Analysis

In the first step of statistical analysis, all continuous data were checked for normality with the Wilk–Shapiro test. Based on the results of this analysis, continuous variables are reported as the mean ± standard deviation (for data with normal distribution) or median and interquartile range (for data with asymmetrical distribution). Categorical variables are reported as the number of cases (N) or % of cases. Differences in continuous variables between the studied groups were first checked with a *t*-test (for a normal distribution) or a u-test (for an asymmetrical one). Differences in categorical variables were assessed with a chi-square test. Multivariable linear regression analysis was performed to assess possible confounding factors. The value of *p* < 0.05 was used as the level of statistical significance. Statistical analysis was performed with the statistical software Statistica version 5.0 (StatSoft, Tula, MS, USA).

## 3. Results

### 3.1. Patients

During the study period, a study group consisting of 39 patients (16 men, 23 women) who met the study enrollment criteria was created. Of these patients, 21 patients had a known T2D (T2D group), and 18 patients had no history of T2D, normal fasting, and post-oral glucose tolerance test levels (non-diabetic/ND/group). The basic demographic data of the enrolled patients are reported in Table 1. Looking at the LVEF, 28 patients presented with pLVEF (15 patients with T2D and 13 ND patients), and 11 patients presented with rLVEF (6 patients in the T2D and 5 patients in the ND group).

### 3.2. Levels of Endothelial Markers at Admission

**Figure 1:**
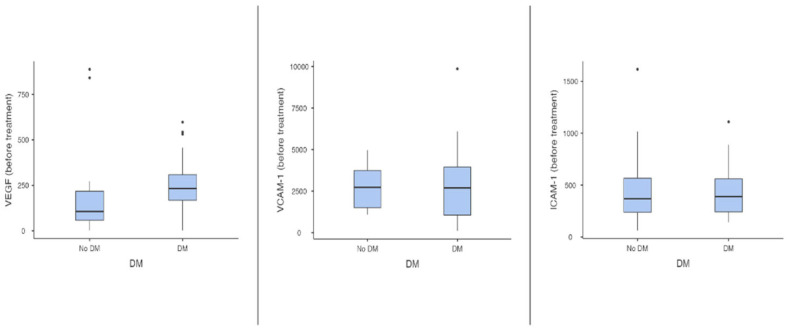
The impact of type 2 diabetes on plasma levels of endothelial markers in patients with acute decompensated heart failure upon admission (ICAM-1—intercellular adhesion molecule 1; VCAM-1—vascular cell adhesion molecule 1; VEGF—vascular endothelial growth factor).

First, the analysis of VEGF levels was performed. The distribution of the marker was asymmetrical. In T2D patients, a median of 233.0 pg/mL (1.7–598 pg/mL) was found compared to 106.0 pg/mL (1.7–888 pg/mL) in ND individuals; the differences reached statistical significance (*p* = 0.04). Second, the analysis of VCAM-1 levels showed a symmetrical distribution of values. There were no significant differences in VCAM-1 levels at admission between T2D and ND patients (2935 ± 2253 ng/mL in the T2D group vs. 2758 ± 1265 ng/mL in the ND group, *p* = 0.73). Third, the analysis of ICAM-1 levels was performed, showing a symmetrical distribution of values. No significant differences between T2D patients and ND patients were found (433 ± 255 ng/mL in the T2D group vs. 486 ± 389 ng/mL in the ND group, *p* = 0.92). These results are displayed in Figure 1.

### 3.3. Levels of Endothelial Markers After Decongestion

**Figure 2:**
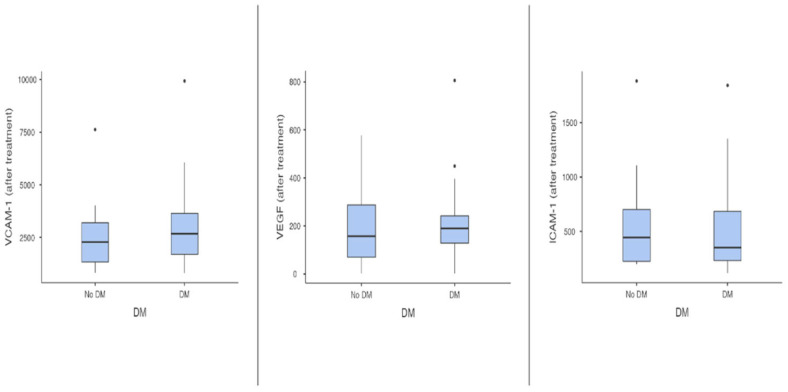
The impact of type 2 diabetes on plasma levels of endothelial markers in patients with acute decompensated heart failure after decongestion (ICAM-1—intercellular adhesion molecule 1; VCAM 1—vascular cell adhesion molecule 1; VEGF—vascular endothelial growth factor).

Subsequently, the analysis of the levels of EM after decongestion (sample 2) was performed. Overall, VEGF (a median of 231 pg/mL before vs. a median of 181 pg/mL after decongestion, *p* = 0.08) and VCAM-1 levels (2692 ± 1843 ng/mL prior vs. 2554 ± 1878 ng/mL after, *p* = 0.97) tended to be lower, and ICAM-1 levels (468 ± 318 ng/mL before vs. 565 ± 440 ng/mL after decongestion, *p* = 0.11) tended to be higher after decongestion. VEGF levels did not differ significantly (*p* = 0.41) between T2D (median value of 190.0 pg/mL, ranging from 1.7 to 806 pg/mL) and ND individuals (median value of 157.0 pg/mL, ranging from 1.7 to 577 pg/mL). Similarly, there were no significant differences in the VCAM-1 (3119 ± 2095 ng/mL in T2D group vs. 2574 ± 1598 ng/mL in ND group, *p* = 0.43) and ICAM-1 (551 ± 449 ng/mL in T2D group vs. 581 ± 442 ng/mL in ND group, *p* = 0.43) levels between T2D and ND patients. These results are displayed in Figure 2.

### 3.4. The Effect of Left Ventricular Ejection Fraction on Levels of Endothelial Markers

Finally, a sub-analysis of EM levels according to LVEF was performed. In patients with pLVEF, there were significant differences in the VEGF levels between T2D patients and those without T2D at admission (205.0 pg/mL, ranging from 1.7 to 598 pg/mL in T2D patients vs. 85.8 pg/mL, with a range from 1.7 to 292 pg/mL in ND group, *p* = 0.02). No significant differences were found in VCAM-1 and ICAM-1 levels between T2D patients and those without T2D at admission, as well as in VEGF, VCAM-1, and ICAM-1 levels after decongestion (Table 2). Similarly, there were no significant differences in the plasma levels of VEGF, VCAM-1, and ICAM-1 between T2D patients and ND patients presenting with rLVEF (Table 3).

### 3.5. Multivariable Regression Analysis

Multivariable linear regression analysis was performed to assess the impact of possible confounding by other factors. In this analysis, we included inflammation, renal function (eGFR and serum creatinine levels), liver function (serum bilirubin levels, alanine-aminotransferase and aspartate-aminotransferase levels), hemoglobin levels, hypertension, smoking status, age, BMI, NT-proBNP levels on admission, and all medications. In this analysis, only the NT-proBNP (*p* = 0.012 for VEGF and *p* = 0.011 for VCAM-1) levels on admission independently predicted VEGF and VCAM-1 levels on admission.

## 4. Discussion

As mentioned, there are limited data on the role of endothelial markers in patients with acute decompensation of chronic HF. In fact, there is only one study addressing this issue. In this study, Iwanek et al. [13] reported that low levels of VEGF (with a median value of 33 pg/mL) in patients admitted for acute HF are associated with more severe signs of congestion and adverse clinical events, with a 1-year mortality rate being the highest in those with low VEGF levels (35% vs. 18% in patients with the highest VEGF levels). This study did not examine other endothelial markers and did not report the T2D status of enrolled patients. In our study, VEGF levels on admission varied from 1.7 pg/mL to 598 pg/mL (with a median of 233.0 pg/mL) in patients with T2D and from 1.7 pg/mL to 888 pg/mL (with a median of 106.0 pg/mL) in patients without T2D. There were significant differences between patients with T2D and ND, mostly seen in those with pLVEF (in patients with rLVEF, only a non-significant trend was observed). After decongestion, there was a slight increase in VEGF levels (to a median value of 157.0 pg/mL) in ND patients; however, this increase was not observed in patients with T2D. For VCAM-1 and ICAM-1 levels, similarly to VEGF levels after decongestion, no significant differences were found between T2D and ND patients at admission, as well as after decongestion. After decongestion, ICAM-1 levels tended to be higher both in T2D and ND patients, but VCAM-1 levels did not change. Unfortunately, there is no other study examining the relationship between T2D and EM levels in patients with acute HF, which currently renders these discrepancies difficult to explain. One can speculate that the changes in endothelial markers might be more potent in identifying decongestion in those without T2D, but there is no satisfactory explanation for this observation at the moment. Undoubtedly, there is a need for future research into the possible role of EM assessments in identifying decongestion. In previously published studies, natriuretic peptides and serum levels of biologically active adrenomedullin have been reported as possible markers of decongestion [28,29], and as already mentioned, one previously published study [13] showed that decreased VEGF levels are associated with a more severe degree of congestion in HF patients; however, as the data gathered so far are very limited, further research is definitely needed to confirm the possible role of VEGF as a marker of congestion in decompensated HF. In fact, the VEGF, VCAM-1, and ICAM-1 levels have been previously associated mostly with vascular complications of T2D [2,3,4,5,6,7,8]. Although endothelial dysfunction is involved in the development of diabetic cardiomyopathy and T2D-related HF [12], the pathology of T2D-related HF is a complex issue and also includes hyperglycemia-induced left ventricular hypertrophy [30], diastolic dysfunction [31] and abnormalities in the metabolism of myocardial cells [32]. Therefore, other mechanisms of HF might play a more important role in T2D-related HF. This could be another possible explanation for the lack of sensitivity of endothelial markers when attempting to identify T2D-related heart failure, which was observed in our study. To summarize, the role of EM assessment in (T2D-related) acute decompensated HF remains indeterminate, is still open for future research, and future studies will be needed before any final conclusions can be adopted.

### Limitations

Our study had several limitations, which must be taken into account when interpreting the results. First, the most important limitation is a low sample size. In addition, the low sample size did not allow further sub-analyses of the results, including the analysis of the impact of HF etiology (ischemic/non-ischemic) on the levels of EM. As this was a pilot study, we did not perform a sample size calculation, and the sample was determined only by the duration period of the study (all patients meeting the study inclusion and not meeting the study exclusion criteria were enrolled). The results of our analysis should be confirmed with larger samples. Second, the decision on the therapy of acute decompensated HF was significantly affected by the attending physician. This could, in theory, cause an unpredictable bias. Third, only patients with acute decompensation of chronic HF were enrolled in our study (patients with “de novo” HF were not enrolled). Thus, the results of our study cannot be applied to all acute HF patients. Finally, only 33% of T2D patients and 17% of ND patients received therapy with sodium–glucose transport protein 2 (SGLT-2) inhibitors upon admission. Although this therapy is currently recommended in chronic HF patients independent of LVEF, it could only be administered to those with non-reduced LVEF (the majority of patients enrolled in our study) in our country starting in August 2023. During the patients’ in-hospital stay, SGLT-2 inhibitor therapy was initiated in the majority of the enrolled patients (with no differences between T2D and ND ones). It might be true that had these agents been used more frequently, this would have had a greater impact on the plasma levels of EMs upon admission [33]. Furthermore, there is a possibility of selection bias, as several patients (see the study exclusion criteria) were excluded from our analysis. Next, it is plausible that we did not control all the possible confounding factors. For example, there was no standardized protocol for diuretic therapy, and the discretion over this therapy was left to the attending physician. This might cause a possible bias (although not likely), as all the patients had met the criteria for decongestion prior to the second blood sampling. To address the issue of other possible confounding factors, we performed a multivariate regression analysis. Nevertheless, this is still an important limitation of our analysis.

As mentioned, there is a need for future research regarding the role of EMs in acute decompensated HF. Unfortunately, due to technical and/or financial limitations, we were not able to assess other EMs, such as the von Willebrand factor (vWF), plasma thrombomodulin, the thrombin-activatable fibrinolysis inhibitor, or E-selectin [34], in our study. It would be of clinical interest to see whether the plasma levels of these markers correlate with congestion/decongestion and clinical courses (length of in-hospital stay, diuretic dose, adverse events, need for HF-related re-admissions, etc.) in patients with T2D and acute decompensated HF. Hopefully, such a study will be performed in the future. Next, the transcript levels of selected EM could be measured by modern PCR to understand if there is any difference in the transcription levels for these EMs. The levels of oxidative stress could also be measured among T2D and ND patients to support the data. We were not able to perform the PCR analysis and the measurement of oxidative stress due to technical and financial difficulties, as this analysis was not, unfortunately, planned when designing our prospective study. Therefore, we did not sample the patients for PCR analysis or oxidative stress examination, as this analysis is extremely costly and could not be covered by our grant support. Hence, this represents another issue that needs to be examined in future research. Furthermore, as reported, EMs seemed to be more significantly changed in T2D patients with pEF (there was only a trend towards differences in those with rEF); however, the number of rEF patients was relatively small in our study. Consequently, a larger study comparing the differences in EM levels between T2D patients with rEF and pEF will be required. Finally, several novel drugs, namely finerenone, semaglutide, and tirzepatide, have been reported as effective for HFpEF or T2D and obesity-related HFpEF [35,36,37,38]. It would be interesting to see how these novel agents affect EM levels in T2D patients with acute decompensated HFpEF. If conducted, such a study would expand our knowledge about the mechanism of action of these novel agents, which is yet to be sufficiently explained. Finally, as previously mentioned, a clear explanation of the possible mechanisms linking the plasma levels of endothelial markers to decongestion is still missing. Although previous studies suggest that these markers can predict decreased lymphatic reserve [39], impaired endothelial barrier function [40], or increased venous stretch induced by systemic congestion [41], further research is needed to address this issue.

## 5. Conclusions

This study identified significant differences in VEGF plasma levels upon admission in T2D patients with acute decompensated HF. Furthermore, the plasma levels of VEGF (in non-diabetic individuals) and ICAM-1 (both in diabetic and non-diabetic individuals) tended to be lower after decongestion. However, these observations come from a pilot study, and the implications of this finding remain unclear. The results advocate future research on the role of EM in predicting decongestion in patients with acute decompensated HF.

## Figures and Tables

**Table 1:** Basic review of selected laboratory parameters, comorbidities, and heart failure therapy in type 2 diabetes and non-diabetic sub-groups of patients with the acute decompensation of chronic heart failure.

	Diabetic Sub-Group	Non-Diabetic Sub-Group
Number of patients (men/women)	21 (7/14)	18 (9/9)
Age	77 (59–88)	75 (56–99)
Beta-blockers at admission/upon release (%)	95/90	83/78
ACE inhibitors, AT1RB and ARNI, at admission/upon release (%)	90/95	78/56
MRA at admission/upon release (%)	67/67	56/72
SGLT2i at admission/upon release (%)	33/62	17/39
CRT at admission/upon release (%)	0/0	0/0
Digoxin at admission/upon release (%)	43/24	61/33
Loop diuretics at admission/upon release (%)	76/90	78/100
BMI (kg/m^2^)	30.3 ± 5.1	25.1 ± 5.0
Serum creatinine (µmol/L)	123.4 ± 77.0	106 ± 35
Calculated GFR using Cocroft–Gault (mL/min/1.73 m^2^)	50.4 ± 22.1	56.1 ± 22.3
ALT (µkat/L)	0.4 ± 0.3	0.4 ± 0.3
AST (µkat/L)	0.5 ± 0.2	0.6 ± 0.3
NT-proBNP (pg/mL)	4100.0 (1411.8–9426.6)	7213.1 (3731.7–13,904)
Hemoglobin (g/L)	116.5 ± 18.1	118.1 ± 20.5
Total serum protein (g/L)	68.7 ± 7.4	63.0 ± 9.4
EF LV (%)	47.2 ± 11.2	47.0 ± 10.1
Etiology of heart failure (%) (ischemic/non-ischemic/indeterminate)	52/33/15	44/39/17
Myocardial revascularization (%)	38	28
History of MI (%)	42	39
Atrial fibrillation (%)	80	72
Arterial hypertension (%)	95	78
Valve disease—moderate-to-severe (%)	57	67
Dyslipidemia (%)	67	22
Duration of diabetes (years)	13 ± 4.5	N/A

ACE—angiotensin-converting enzyme; AT1RB—angiotensin 1 receptor blocker; ARNI—angiotensin receptor/neprilysin inhibitor; MRA—mineralocorticoid receptor antagonist; SGLT2i—sodium–glucose cotransporter-2 inhibitor; CRT—cardiac resynchronization therapy; BMI—body mass index; GFR—glomerular filtration rate; ALT—alanine aminotransferase; AST—aspartate aminotransferase; NT-proBNP—N-terminal prohormone of brain natriuretic peptide; EF LV—ejection fraction of left ventricle; MI—myocardial infarction; N/A—not applicable.

**Table 2:** The impact of type 2 diabetes on plasma levels of endothelial markers in patients with preserved left ventricular ejection fraction.

Patients with pLVEF	T2D	ND	Significance (*p* Value)
Number of patients	15	13	N/A
VEGF levels pg/mL (sample 1)	205.0 (1.7–598)	85.8 (1.7–292)	0.02
VCAM-1 levels ng/mL (sample 1)	3188.0 ± 2524.0	2666.0 ± 1176.0	0.96
ICAM-1 levels ng/mL (sample 1)	476.0 ± 285.0	396.0 ± 276.0	0.36
VEGF levels pg/mL (sample 2)	186.0 (42–264)	159.9 (1.7–577)	0.61
VCAM-1 levels ng/mL (sample 2)	3431.0 ± 2157.0	2809.0 ± 1771.0	0.36
ICAM-1 levels ng/mL (sample 2)	608.0 ± 487.0	542.0 ± 463.0	0.72

ICAM-1—intercellular adhesion molecule 1; ND—patients without type 2 diabetes; N/A—not applicable; pLVEF—preserved left ventricular ejection fraction; T2D—patients with type 2 diabetes; VCAM-1—vascular cell adhesion molecule; VEGF—vascular endothelial growth factor.

**Table 3:** The impact of type 2 diabetes on plasma levels of endothelial markers in patients with reduced left ventricular ejection fraction.

Patients with rLVEF	T2D	ND	Significance (*p* Value)
Number of patients	6	5	N/A
VEGF levels pg/mL (sample 1)	281.0 (57.0–553.0)	288.4 (1.7–888.0)	0.54
VCAM-1 levels ng/mL (sample 1)	2495.0 ± 1342.0	2652.0 ± 1598.0	0.45
ICAM-1 levels ng/mL (sample 1)	328.0 ± 114.0	498.0 ± 252.0	0.13
VEGF levels pg/mL (sample 2)	293.0 (1.7–806.0)	77.2 (1.7–82.2)	0.30
VCAM-1 levels ng/mL (sample 2)	1741.0 ± 1071.0	2196.0 ± 895.0	0.79
ICAM-1 levels ng/mL (sample 2)	240.0 ± 134.0	904.0 ± 422.0	0.27

ICAM-1—intercellular adhesion molecule 1; ND—patients without type 2 diabetes; N/A—not applicable; rLVEF—preserved left ventricular ejection fraction; T2D—patients with type 2 diabetes; VCAM-1—vascular cell adhesion molecule; VEGF—vascular endothelial growth factor.

## Data Availability

All the source data are available from the corresponding author upon reasonable request.
